# Case of Excision of Left Superior Maxillary and Molar Bones and One-Half of the Nose

**Published:** 1874-04

**Authors:** M. Metivier

**Affiliations:** HOLYOKE, MASS.


### ARTICLE IX.

### *Case of Excision of the left Superior Maxillary and Malar Bones, and one-half of the Nose.*

BY M. METIVIER, HOLYOKE, MASS.

A few weeks since, Dr. Metivier operated on a woman suffering a large cancerous tumour, which involved the left cheek, and left side of the nose, together with the Superior Maxillary and Malar bones, on the same side. As a large portion of the integument and tissues below the eye had been removed by ulceration, and a large gap was left, it was not possible to make a flap in the usual way; the following plan was therefore adopted. The patient was seateR in a chair, and put slightly under the influence of chloroform, the head leaning well forward, to prevent the blood from running into the throat. An incision was then made, beginning on the middle of the nose, at the nasal process of the superior Maxillary bone, and was carried downwards, dividing the nose at the edge of the septum, and opening up the nostril. It was then carried down, dividing the upper lip in the middle line, into the mouth. A second incision was made from the zygoma, horizontal to the superior border of the Malar bone, and on the margin of the orbital plate. This incision curved upwards. A third incision was then made, beginning at the external angular process of the frontal bone, and running obliquely downwards to the lower margin of the nostril, connecting with the first incision, and leaving a large triangular portion of integument to be excised with the tumour. Several arteries required to be tied at this stage of the operation. The soft parts of the hard palate were then divided, and a tooth drawn; the alveolar process was sawed through, and the palatine and orbital plates divided with the bone pliers. The saw was again used to divide the Malar bone, and the Zygomatic process. A few sweeps of the knife, with strong depression, brought down the mass out of its place, leaving a large hole. The patient was roused from under the influence of chloroform several times during the operation, in order that she might clear the throat from clots of blood, and also that she might take a stimulant. The bleeding was slight, not amounting to more than three ounces. In order to replace the lost integuments which had been excised with the tumour, the incision on the zygoma and the dissection of the integuments was extended almost to the ear. The flap was stretched and attached to the root of the nose by interrupted sutures. It was cut of the proper shade to adopt itself to form the lost half of the nose, and then was attached to the whole length of the side of the nose, and also the lip. It was also fixed to the lower eyelid by several sutures. The cheek was filled up with a large piece of linen soaked in carbolic acid and oil, and carbolic acid water dressing was applied externally to the whole wound. The patient walked to bed after the operation, saying that she was not weak, and did not suffer any pain. In one month's time the wound had healed, leaving scarcely any disfigurement, much to the satisfaction of the patient, who could not be persuaded that the disease would certainly recur.—*Canada Med. and Surg. Journal.*

